# Fatal Disulfiram-Ethanol Reaction in a Patient With Preexisting Cardiac Comorbidities: A Case Report

**DOI:** 10.7759/cureus.83229

**Published:** 2025-04-29

**Authors:** Thyanesh G, Sibi Vijaya Kumar, Venkatesan M, Divya Madhala

**Affiliations:** 1 Forensic Medicine and Toxicology, Sri Ramachandra Institute of Higher Education and Research, Chennai, IND; 2 Pathology, Sri Ramachandra Institute of Higher Education and Research, Chennai, IND

**Keywords:** acute tubular necrosis, alcohol dependence, cardiac comorbidities, disulfiram-ethanol reaction, forensic pathology

## Abstract

Disulfiram is widely used in the management of alcohol dependence; however, its interaction with ethanol can result in severe and life-threatening complications. This case report highlights the fatal outcome of a disulfiram-ethanol reaction (DER) in a patient with preexisting cardiac comorbidities. A 51-year-old male, recently discharged from a rehabilitation center after five months of treatment, resumed alcohol consumption despite strict medical warnings. This led to a severe DER, culminating in hypotensive shock and multi-organ failure, ultimately proving fatal despite aggressive medical intervention. Autopsy findings revealed acute tubular necrosis (ATN) secondary to persistent hypotension, exacerbated by preexisting dilated cardiomyopathy and previous myocardial infarction. This case underscores the risks of disulfiram therapy in patients with significant cardiovascular disease and highlights the need for alternative pharmacologic strategies to prevent alcohol relapse in high-risk individuals.

## Introduction

Disulfiram has been a cornerstone in the pharmacological management of alcohol use disorder due to its ability to create an aversive reaction upon ethanol ingestion. Disulfiram has been used in alcohol dependence treatment since its FDA approval in 1951, making it one of the oldest pharmacotherapies available for this condition [1]. Disulfiram is still frequently given, even though newer drugs have entered the market; research estimates that 10%-15% of individuals receiving pharmacological treatment for alcohol use disorder are prescribed this medication [2]. Disulfiram's capacity to inhibit aldehyde dehydrogenase, with an 80% inhibition rate within 12 hours of dosing, provides the physiological basis for its efficacy. This results in acetaldehyde concentrations that are 5-10 times higher than usual when alcohol is consumed [3].

Disulfiram acts by irreversibly inhibiting aldehyde dehydrogenase, leading to the accumulation of acetaldehyde, which produces severe symptoms such as flushing, palpitations, hypotension, arrhythmias, myocardial infarction, and cardiovascular collapse [4-6]. Even small amounts of alcohol can cause major reactions in sensitive people, demonstrating the dose-dependent nature of these reactions [7]. Disulfiram is still used in certain cases, as long as the patients are closely followed, even though it is generally contraindicated for those with severe heart disease, kidney failure, or cerebrovascular diseases [8].

Following a disulfiram-ethanol reaction (DER), a number of investigations have documented abrupt circulatory collapse, myocardial ischemia, and acute renal damage; the results vary depending on the underlying comorbidities and the promptness of medical response [9]. The literature indicates that even small quantities of alcohol, including those found in certain foods and medications, can trigger a disulfiram reaction with potentially severe consequences [10]. The present case illustrates the devastating impact of DER in a patient with known cardiac disease, despite medical adherence to standard disulfiram therapy protocols, and highlights the fatal progression of DER in a high-risk individual, underscoring the urgent need for alternative pharmacologic approaches in alcohol relapse prevention for patients with preexisting cardiovascular disease.

## Case presentation

Patient history

A 51-year-old male, recently discharged from an alcohol rehabilitation center following five months of abstinence, presented to the emergency room (ER) with severe respiratory distress, frothing from the mouth, and altered sensorium. He had been prescribed disulfiram therapy upon discharge, with strict instructions to avoid alcohol consumption. However, after resuming alcohol intake, he developed a severe systemic reaction within hours.

The patient had multiple comorbidities, including dilated cardiomyopathy with a reduced ejection fraction (38%), type 2 diabetes mellitus, and a history of a previous inferior wall myocardial infarction in 2015. Upon arrival at the ER, he was profoundly hypotensive (BP: 60/40 mmHg) and exhibited signs of severe metabolic acidosis (pH: 7.172 and bicarbonate: 15.3 mmol/L), respiratory distress, and altered mental status. Laboratory investigations revealed acute kidney injury (creatinine: 4.2 mg/dL) and an elevated troponin T level (9.2 ng/mL), indicative of significant myocardial stress (Table 1).

**Table 1:** Laboratory test parameters Normal/Reference Range: [11]

Parameter	Sample Type	Findings (This Case)	Normal/Reference Range
pH	Blood	7.172	7.35 - 7.45
Bicarbonate (HCO₃)	Blood	15.3 mmol/L	22 - 28 mmol/L
Creatinine (Cr)	Blood	4.2 mg/dL	0.7 - 1.3 mg/dL
Troponin T (cTnT)	Blood	9.2 ng/mL	<0.01 ng/mL

Diagnostic evaluation included an echocardiogram, which showed a further reduced ejection fraction (32%), mild mitral regurgitation, and no evidence of pericardial effusion or intracardiac thrombus. A computed tomography (CT) brain scan ruled out acute infarction or hemorrhage.

The patient underwent aggressive medical intervention. Cardiac support included synchronized direct current (DC) cardioversion for ventricular tachycardia, along with intravenous amiodarone and norepinephrine infusion to manage persistent hypotension. Renal support was initiated with continuous renal replacement therapy (CRRT) to address worsening metabolic acidosis. Due to respiratory failure, he was intubated and placed on mechanical ventilation.

The patient's condition progressively worsened despite aggressive care, leading to bradycardia, asystole, and refractory shock. Although advanced cardiac life support (ACLS) was started, spontaneous circulation could not be restored. After three days of critical care, the patient was declared deceased, and an autopsy was conducted.

Autopsy and histopathology findings

**Figure 1:**
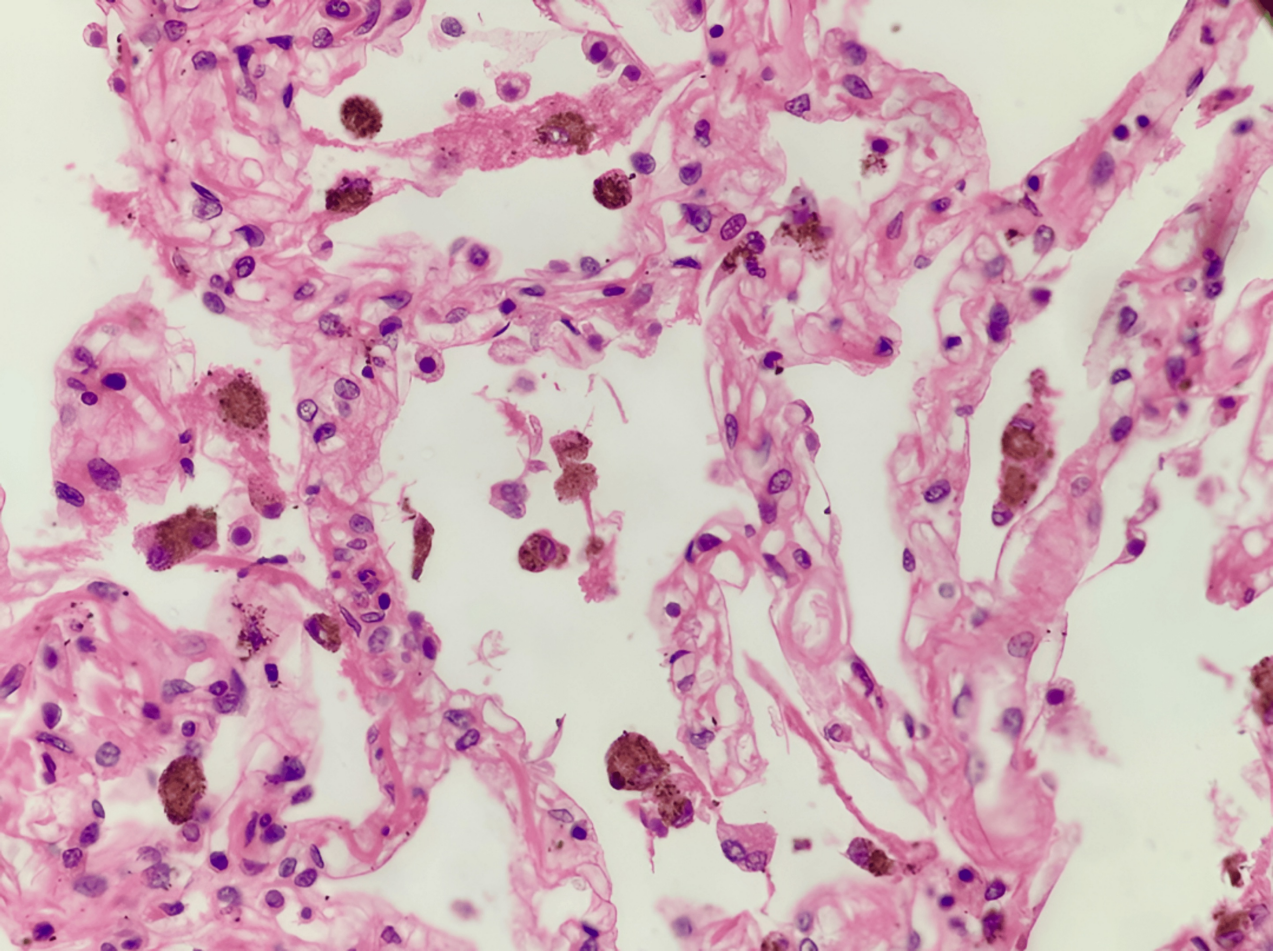
Section from lung shows dilated alveoli with hemosiderin-laden macrophages (H&E, 20x)

**Figure 2:**
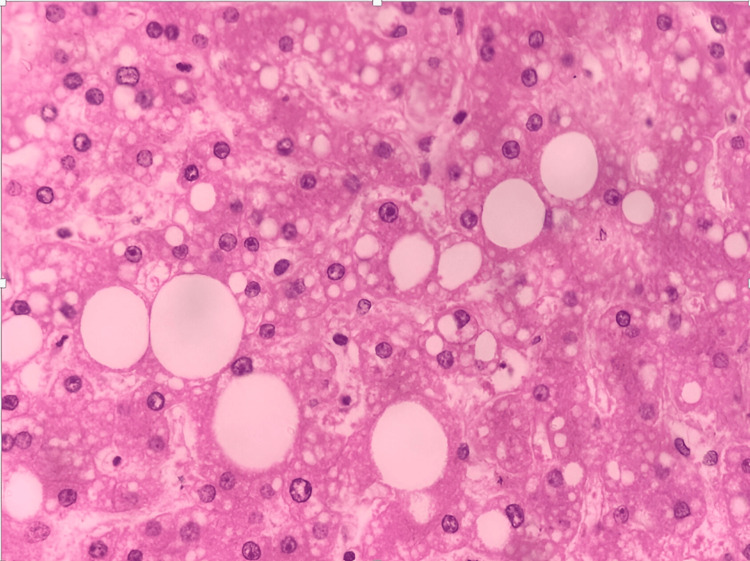
Section from liver shows features of microvesicular and macrovesicular steatosis (H&E, 40x)

**Figure 3:**
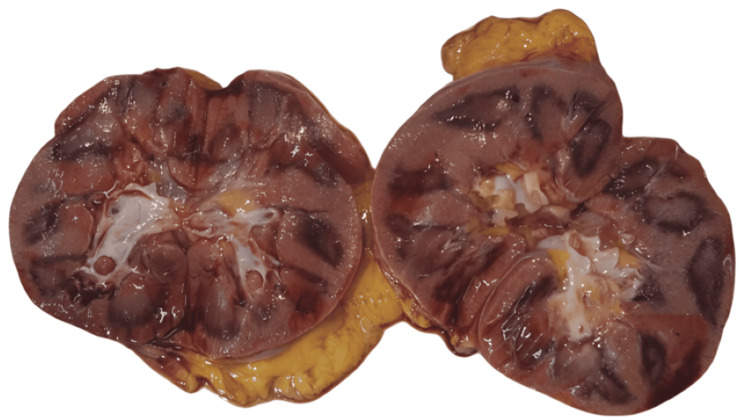
Image showing hemorrhagic kidneys with an ill-defined corticomedullary junction

**Figure 4:**
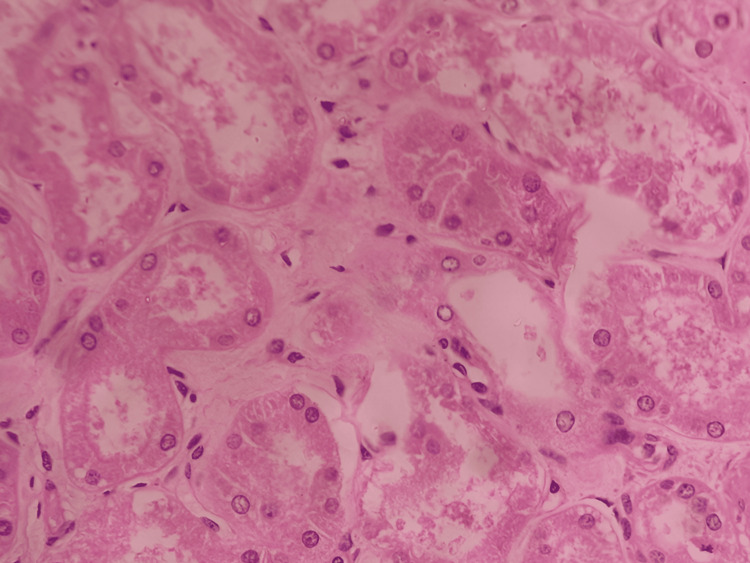
Section from kidney shows acute tubular necrosis (H&E, 20x)

**Figure 5:**
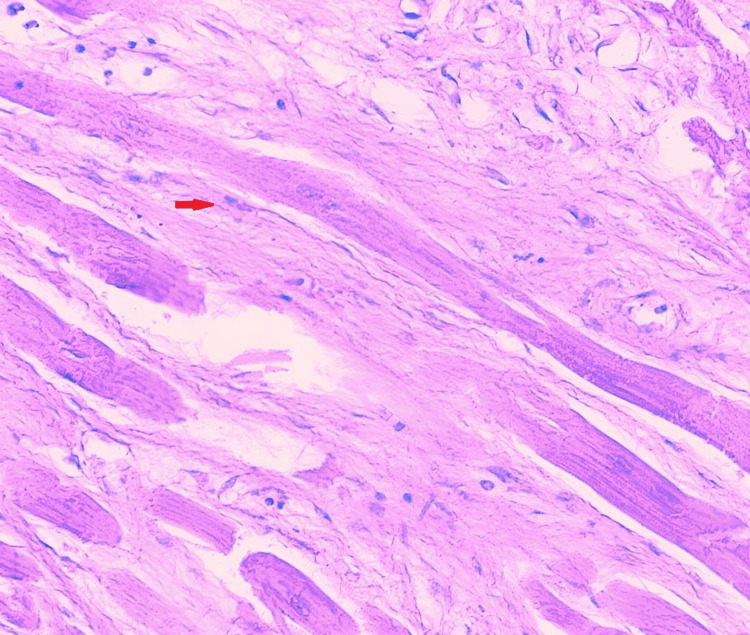
Section of heart shows replacement fibrosis (red arrow; H&E, 40x)

**Figure 6:**
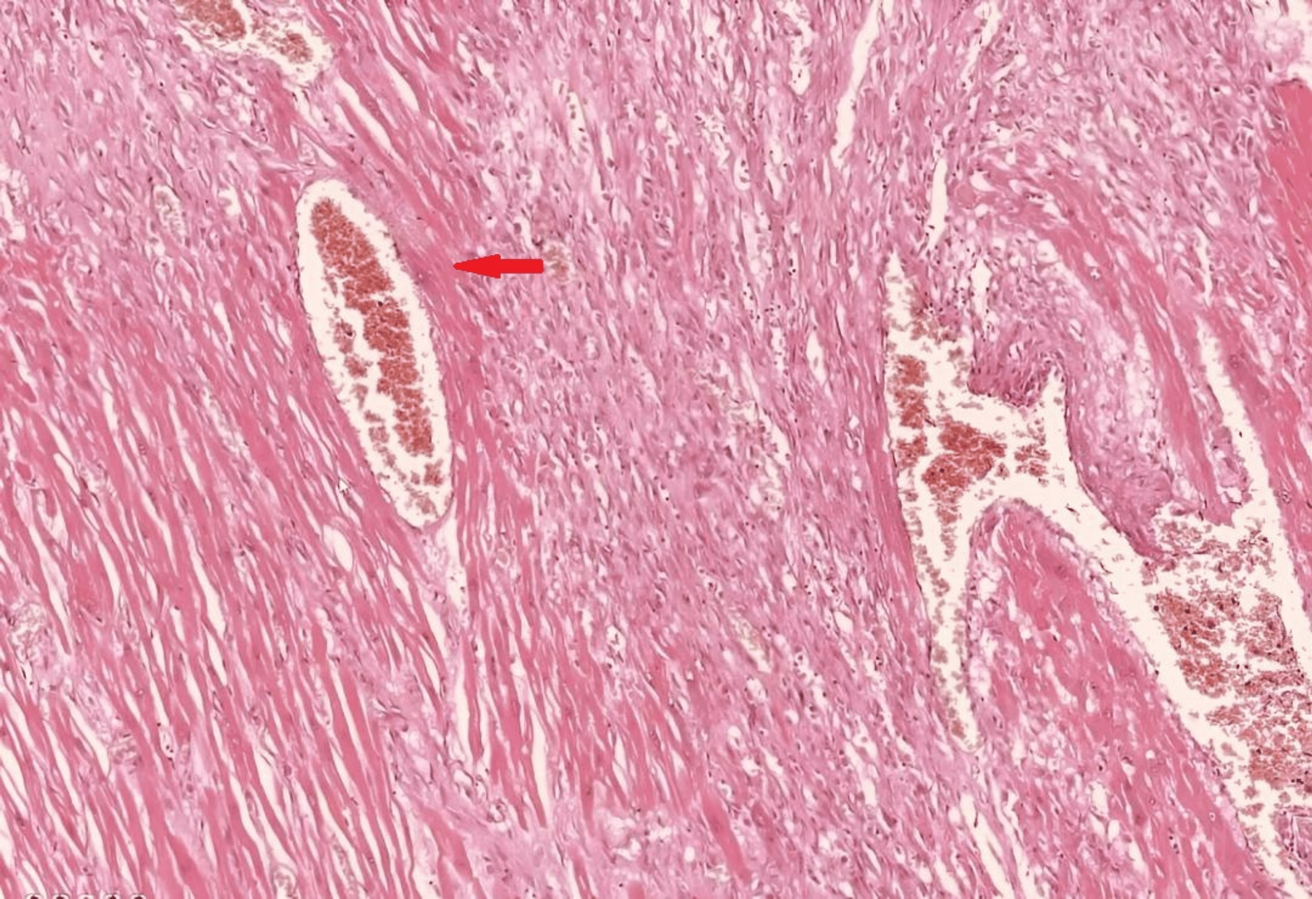
Section from heart shows myocardial hypertrophy (red arrow; H&E, 40x)

A gross examination revealed bilateral pulmonary edema with consolidation in the lower lobes, and histopathological examination showed dilated alveoli with hemosiderin-laden macrophages (Figure 1). The peritoneal cavity contained approximately 200 mL of straw-colored fluid. The liver exhibited yellowish discoloration, suggestive of fatty changes, and histopathological examination confirmed features of alcoholic liver disease with both macrovesicular and microvesicular steatosis (Figure 2). The kidneys appeared hemorrhagic, with an ill-defined corticomedullary junction (Figure 3), and histopathology showed acute tubular necrosis (ATN) (Figure 4). The heart was enlarged (430 g) with biventricular dilatation, consistent with the patient's known dilated cardiomyopathy. An area of fibrosis was identified in the inferior wall, corresponding with the patient's previous myocardial infarction history. Histopathological confirmation of this old infarct showed replacement fibrosis (Figure 5) and myocyte hypertrophy (Figure 6) in surrounding viable myocardium. No evidence of recent/acute myocardial infarction was observed. Though comprehensive cardiac histopathology was not performed, the gross findings were consistent with the patient's known cardiac history. Additionally, the intestines exhibited signs of inflammation on gross examination. The brain appeared congested, with petechial hemorrhages noted on the surface.

Chemical analysis for alcohol was performed during the autopsy; however, it returned negative, likely attributable to the metabolic clearance of alcohol during the prolonged hospitalization period prior to death. Despite the negative alcohol detection, the clinical course, sequence of systemic complications, and characteristic features were highly consistent with a severe DER. Based on the documented clinical events - including profound hypotension, progressive cardiac dysfunction, refractory metabolic acidosis, acute kidney injury, multi-organ failure, and the absence of other alternative etiologies - the cause of death was determined to be cardiogenic shock with multi-organ failure, precipitated by a DER in a patient with significant preexisting cardiac disease.

## Discussion

Disulfiram's inhibition of aldehyde dehydrogenase leads to excessive acetaldehyde accumulation, causing hypotension, cardiovascular instability, and organ damage [12]. Studies have shown that DER can lead to acute cardiac complications, particularly in individuals with pre-existing heart failure or ischemic heart disease [8,13,14].

Regarding the threshold for DERs, studies indicate that blood alcohol concentrations as low as 5-10 mg/dL can trigger symptoms in patients taking therapeutic doses of disulfiram [15]. The severity of the reaction varies depending on individual susceptibility, disulfiram dosage, and ethanol intake [8]. Reports indicate that even small amounts of alcohol can precipitate a fatal reaction in high-risk patients, particularly those with preexisting myocardial dysfunction. In such cases, cardiogenic shock and refractory hypotension become inevitable complications that contribute to multi-organ failure [16]. Although blood alcohol testing was negative in our patient - due to the three-day hospitalization period prior to death - the clinical presentation was classic for a severe DER.

The macrovesicular and microvesicular steatosis observed in our patient's liver (Figure 1) represents a common finding in chronic alcohol users and contributes to our understanding of the patient's overall clinical picture. Macrovesicular steatosis is characterized by large lipid droplets that displace the nucleus to the cell periphery, and it typically results from chronic alcohol consumption, obesity, and metabolic syndrome [17]. While not directly related to the acute cause of death, this hepatic pathology indicates long-term alcohol abuse and potentially reduced liver function, which may have impaired the patient's ability to metabolize both alcohol and disulfiram effectively. Compromised hepatic function can lead to higher circulating levels of acetaldehyde during a DER, potentially exacerbating the cardiovascular effects [18].

The myocardial effects of disulfiram are further complicated by research indicating that acetaldehyde directly impairs cardiac contractility through calcium handling disruption in cardiomyocytes [19]. This mechanism is particularly relevant to our case, as it explains how DER can precipitate fatal cardiac dysfunction without producing new structural cardiac lesions identifiable on histopathology. Studies have demonstrated that acetaldehyde directly interferes with excitation-contraction coupling in cardiac myocytes through inhibition of sarcolemmal calcium transport and disruption of calcium sequestration by the sarcoplasmic reticulum [20] - mechanisms that would disproportionately affect a heart already compromised by dilated cardiomyopathy.

Additionally, the systemic vasodilation triggered by acetaldehyde accumulation creates a profound mismatch between vascular tone and cardiac output, which is particularly dangerous in patients with preexisting myocardial dysfunction [21]. Recent studies using echocardiographic assessments during controlled DER have demonstrated acute reductions in ejection fraction of up to 15%, even in patients without baseline cardiac disease [22]. In our patient, the reduction from a baseline ejection fraction of 38% to 32% during hospitalization - while seemingly modest - represented a critical deterioration in an already compromised heart, pushing the patient below the threshold of compensatory reserve and into cardiogenic shock.

The ATN observed in our patient highlights the complex interaction between hemodynamic instability and direct nephrotoxicity in DERs. Several case reports have documented the progression of hemodynamic instability - including refractory hypotension and shock - in patients experiencing a DER [9,23]. Given that ATN is a well-recognized consequence of prolonged hypotension, it is plausible that DER-induced circulatory collapse can contribute to ischemic renal injury in susceptible individuals. Pathophysiological studies indicate that metabolic acidosis and sustained hypotension in shock states further exacerbate renal dysfunction, potentially leading to irreversible damage [24].

The intestinal inflammation and cerebral congestion with petechial hemorrhages observed at autopsy represent additional manifestations of multi-organ dysfunction in this case. Intestinal inflammation likely reflects a combination of direct acetaldehyde toxicity and splanchnic hypoperfusion secondary to profound hypotension [25]. Similarly, the cerebral findings are consistent with disrupted cerebrovascular autoregulation during shock, potentially compounded by acetaldehyde's known neurotoxic effects [26]. These observations further support our conclusion that DER precipitated widespread systemic dysfunction beyond the primary cardiac insult, contributing to the fatal outcome.

Recent clinical guidelines have emphasized the importance of careful patient selection for disulfiram therapy, with particular caution in those with cardiovascular disease [27]. Alternative pharmacologic strategies, such as naltrexone and acamprosate, have been suggested for individuals with cardiac comorbidities, as these medications do not cause cardiovascular instability [8]. Naltrexone, an opioid receptor antagonist, reduces alcohol cravings without causing adverse reactions upon alcohol consumption, while acamprosate acts on glutamate and GABA (gamma-aminobutyric acid) neurotransmission to reduce withdrawal symptoms and cravings [28]. Given the high mortality risk associated with DER in cardiac patients, clinicians should consider personalized therapy options and avoid disulfiram in high-risk individuals.

Forensic implications of this case highlight the importance of medicolegal awareness regarding DER-associated fatalities, especially in patients under supervised rehabilitation programs. Clinicians prescribing disulfiram must ensure strict patient education and monitoring, particularly for those with high-risk cardiovascular conditions, and explore safer alternatives to minimize mortality risk. The case also underscores the need for comprehensive screening protocols to identify patients with latent or undiagnosed cardiac conditions, who may be at elevated risk for severe DER complications.

## Conclusions

This case underscores the potentially fatal outcome of DER in patients with preexisting cardiovascular disease, despite adherence to treatment protocols. The primary cause of death was cardiogenic shock with multi-organ failure, directly precipitated by DER in a patient with compromised cardiac function. Notably, this occurred through functional impairment, with acetaldehyde-induced depression of myocardial contractility and peripheral vasodilation being the primary mechanisms. Severe hypotension led to ATN and subsequent mortality, demonstrating the particular vulnerability of patients with cardiac comorbidities to the hemodynamic effects of DER. The findings emphasize the importance of individualized pharmacologic therapy, recommending alternative agents such as naltrexone or acamprosate for high-risk patients. Awareness of DER-related complications is crucial for preventing fatal outcomes in alcohol dependence management.
